# Macrophage migration inhibitory factor as a diagnostic and predictive biomarker in sepsis: meta-analysis of clinical trials

**DOI:** 10.1038/s41598-021-87613-0

**Published:** 2021-04-13

**Authors:** Janos Toldi, David Nemeth, Peter Hegyi, Zsolt Molnar, Margit Solymar, Nelli Farkas, Hussain Alizadeh, Zoltan Rumbus, Eszter Pakai, Andras Garami

**Affiliations:** 1 Department of Thermophysiology, Institute for Translational Medicine, Medical School, University of Pecs, Pecs, Hungary; 2 Department of Anesthesiology and Intensive Care, Medical School, University of Pecs, Pecs, Hungary; 3 Institute for Translational Medicine, Medical School and Szentagothai Research Centre, University of Pecs, Pecs, Hungary; 4 Department of Anesthesiology and Intensive Therapy, Poznan University of Medical Sciences, Poznan, Poland; 5 Institute of Bioanalysis, Medical School, University of Pecs, Pecs, Hungary; 6 Division of Hematology, First Department of Medicine, University of Pecs, Pecs, Hungary

**Keywords:** Diagnostic markers, Predictive markers, Prognostic markers, Sepsis

## Abstract

The hunt for useful sepsis biomarkers is ongoing. Macrophage migration inhibitory factor (MIF) was implicated as a biomarker in sepsis, but its diagnostic and prognostic value has remained unclear in human studies. Here, we aimed at clarifying the value of MIF as a sepsis biomarker with the meta-analysis of clinical trials. PubMed, EMBASE, and Cochrane Central Register of Controlled Trials databases were searched until December 2019. From the included studies, blood MIF levels and indicators of disease severity were extracted in septic and control patient groups. Twenty-one eligible studies were identified, including data from 1876 subjects (of which 1206 had sepsis). In the septic patients, blood MIF levels were significantly higher than in healthy controls with a standardized mean difference (SMD) of 1.47 (95% confidence interval, CI: 0.96–1.97; *p* < 0.001) and also higher than in patient groups with nonseptic systemic inflammation (SMD = 0.94; CI: 0.51–1.38; *p* < 0.001). Markedly greater elevation in blood MIF level was found in the more severe forms of sepsis and in nonsurvivors than in less severe forms and in survivors with SMDs of 0.84 (CI: 0.45–1.24) and 0.75 (CI: 0.40–1.11), respectively (*p* < 0.001 for both). In conclusion, blood MIF level is more elevated in systemic inflammation caused by infection (i.e., sepsis) compared to noninfectious causes. In more severe forms of sepsis, including fatal outcome, MIF levels are higher than in less severe forms. These results suggest that MIF can be a valuable diagnostic and prognostic biomarker in sepsis given that well-designed clinical trials validate our findings.

## Introduction

Sepsis, a form of systemic inflammation, is defined as life-threatening organ dysfunction caused by dysregulated host response to infection^1^. Even nowadays, sepsis and related diseases represent a major challenge for the healthcare system. According to a novel analysis of cause-of-death data from 109 million records in the Global Burden of Diseases, Injuries, and Risk Factors Study, nearly 49 million incident cases of sepsis could be estimated worldwide and 11 million sepsis-related deaths were reported^2^. In a cohort from 6 hospitals in the US, sepsis was present in more than half of the hospitalizations and accounted for the highest ratio (35%) among the causes of death^3^. While there was some evidence of a trend towards decreasing mortality rates in septic patients over the last decade, a continuous decline in mortality was not observed among patients with sepsis or septic shock in a recent systematic review^4^. These data warrant for the need of better sepsis management, which could be facilitated by improved diagnostic and prognostic tools.

In spite of the desperate need for reliable biomarkers in sepsis, according to the Sepsis-3 definition consensus, the novel candidates require further validation before they can be incorporated into the clinical practice^1^. In 2010, an electronic search identified 178 sepsis-related biomarkers, but none of them was found eligible for routine use in clinical practice^5^. According to a current review by the same group^6^, the list of potential biomarkers in sepsis has expanded, and in 2020 it included more than 250 substances, but only a few of them were evaluated in a large patient population or in repeated studies, which still limits their clinical usability.

Macrophage migration inhibitory factor (MIF), a mediator of the innate immune system, is involved in many inflammatory processes and related disorders, including obesity and diabetes mellitus^7,8^, autoimmune disorders^7,9^, and cancer^7,10^. Besides the role of MIF in chronic inflammation, as a proinflammatory cytokine, it is rapidly released into the bloodstream in different forms of acute systemic inflammation^11^. The causes of acute systemic inflammation can be diverse, including diseases induced by microbial pathogens (e.g., sepsis, septic shock), as well as, noninfectious illnesses due to stress, autoimmune reaction, trauma, surgery, burns, etc. Increased blood MIF levels were reported in forms of acute systemic inflammation originating from both infectious and noninfectious etiologies^7^, but it has remained questionable whether the magnitude of the increase is similar or different in the two forms, therefore, if MIF can be used as a diagnostic tool in sepsis. A similar increase in MIF levels was observed in patients with systemic inflammation of septic and nonseptic (i.e., caused by major surgery) origin compared to the healthy controls^12^, suggesting that MIF may serve as a biomarker for critical illness without the ability to differentiate between infectious and noninfectious causes. However, in other studies, MIF levels were markedly higher in sepsis than in patients with other forms of systemic inflammation^13–15^, indicating that MIF can be used as a diagnostic biomarker in sepsis.

The prognostic value of MIF has also remained controversial. On the one hand, high serum levels of MIF were found in septic patients and even higher MIF levels in patients with septic shock, though the difference was not statistically significant (*p* = 0.3)^16^. Not significantly higher MIF levels were also reported in septic patients with lung complications compared to those without it^13^. On the other hand, no significant correlation was found between serum MIF levels and sepsis severity or mortality^17^. Further complicating the issue, circulating MIF levels did not differ between sepsis survivors and nonsurvivors in one study^18^, whereas nonsurvivors had significantly higher MIF levels in another study^13^.

In the present meta-analysis, we aimed at studying the diagnostic and prognostic value of blood MIF levels in sepsis by analyzing the currently available published data in humans.

## Methods

Our meta-analysis was conducted in accordance with the guidelines of the PRISMA (Preferred Reporting Items for Systematic Reviews and Meta-Analysis) statement^19^ (Supplementary Table S1). The question of our analysis was defined in the PICO [Patients, Indicator, Comparison, Outcome] format: in adult septic patients, we aimed at assessing the biomarker role of MIF in the diagnosis and prognosis of the disease. This meta-analysis has been registered with PROSPERO (CRD42020139137).

### Search strategy

We searched the PubMed, EMBASE, and CENTRAL (Cochrane Central Register of Controlled Trials) databases for original human studies without time period limitations. The following search term was used: ("macrophage migration inhibitory factor" OR MIF) AND (sepsis OR septic). As in our previous meta-analysis of sepsis^20^, publications reporting immunosuppressive conditions (e.g., transplantation, HIV infection) were not included in the current analysis. Similarly to our past studies^20,21^, the search was conducted separately by two authors (JT, AG), who also assessed study eligibility and extracted data from the selected studies independently. Disagreements were resolved by consensus, with the help of a third party (ZR).

### Study selection, data extraction, and risk of bias assessment

The titles and abstracts of the publications identified by the literature search were screened, and the full texts of potentially eligible articles were obtained. We included studies which reported blood MIF levels in two or more different patient groups, at least one of which groups consisted of septic patients. For analysis of the prognostic value, an indication of disease severity or outcome (e.g., mortality rate) was also required for the groups. From all included articles we extracted the country of origin, characteristics of the patient populations (sample size, sex ratio, age, severity score, mortality), and the reported blood MIF level values of the patient groups with the corresponding indicator of standard deviation (SD). The extracted values were converted to mean and SD unless specified otherwise. Different patient groups within a study (e.g., survivor vs. nonsurvivor, septic vs. nonseptic systemic inflammation) were extracted separately.

We assessed the quality of each study included in the meta-analysis by using the Newcastle–Ottawa Scale^22^ (Supplementary Table S2).

### Statistical analysis

For each included study, we calculated the difference between the blood MIF level of a septic patient group and that of another septic group or a control group. For all groups, the means were standardized (based on variances) to obtain standardized mean differences (SMDs). For standardization, the means were divided by their corresponding SD values, which was required because the different MIF measuring methods could result in different variances among the study groups and influence the results. The SMDs with 95% confidence intervals (CIs) were calculated by using the random effect model by DerSimonian and Laird^23^, and then compared using standard meta-analysis tools (i.e., forest plot).

In accordance with the Cochrane Handbook for Systematic Reviews^24^, between-study heterogeneity was tested with *I*^2^ statistical test, where *I*^2^ is the proportion of total variation attributable to between-study variability (an *I*^2^ value of more than 50% was considered as an indication of substantial heterogeneity). The presence of publication bias was determined by visual inspection of funnel plots (Supplementary Figs. S1-S4) for the lack of asymmetry and evaluated quantitatively by Egger’s test (*p* < 0.1 indicating publication bias). Sensitivity analysis (i.e., iteratively omitting one study from the analyses and recalculating SMD to investigate the impact of the individual study on the summary estimate) was performed to test the impact of the individual studies. The meta-analyses were performed with Comprehensive Meta-Analysis (version 3.3; Biostat, Engelwood, MJ, USA) software.

A receiver operating characteristic (ROC) curve was constructed to evaluate the diagnostic performance of blood MIF levels in sepsis. For that, individual blood MIF level data of septic patients and healthy controls were extracted with WebPlotDigitizer application from eligible papers^25–27^, which presented the data in figures with linear scales. The area under the ROC curve (AUC) was calculated to assess the accuracy of blood MIF level measurement as a diagnostic test in sepsis. Within the range of 0.5 (no diagnostic ability) to 1.0 (perfect diagnostic ability), a higher AUC indicates better performance of a test. ROC curve analysis was performed using IBM SPSS Statistics for Windows, version 26 (IBM Corporation, Armonk, NY, USA).

## Results

### Study selection, characteristics, and quality

**Figure 1:**
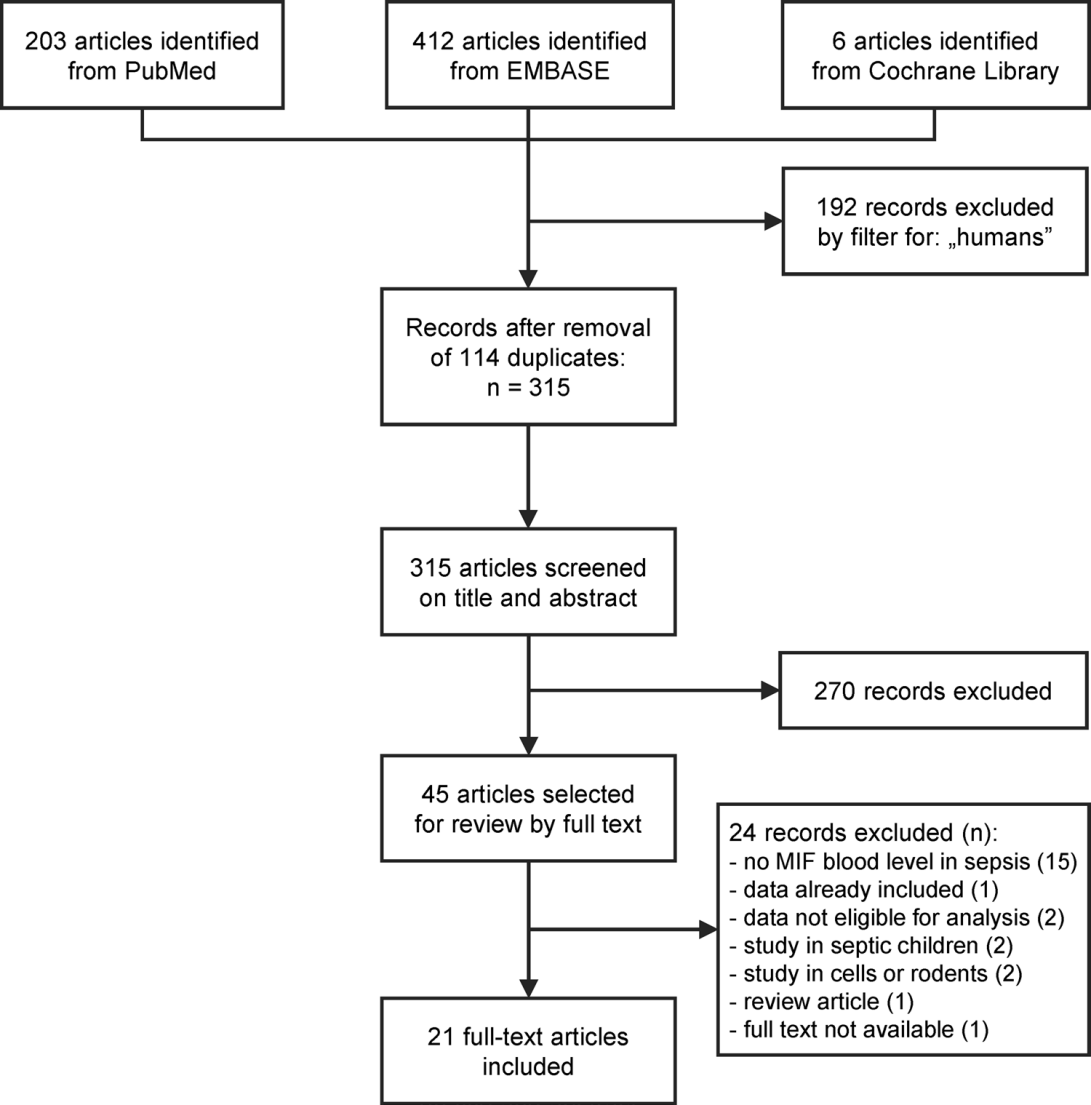
Flowchart of study selection and inclusion.

The flow chart of the study selection is presented in Fig. 1. Until December 2019, the electronic literature search identified altogether 621 studies from the PubMed, EMBASE, and CENTRAL databases. After enabling filters for human studies and removal of duplicates, 315 articles remained, which were screened on title and abstract for inclusion criteria. As a result, the full texts of 45 articles were obtained, out of which 21 publications were found eligible for statistical analysis^12–18,25–38^, including data from a total of 1876 human subjects. The studied groups consisted of 1206 septic patients, 134 patients with noninfectious systemic inflammation, and 536 healthy controls (i.e., subjects without known systemic inflammation). The study characteristics are presented in Table 1.

**Table 1:** Characteristics of participants in the studies included in the meta-analysis.

Study report [number in list of references]	Country	Study population	Population subgroups	N (males)	Mean years of age (SD)	Mean severity score (SD)	Deaths N (%)
Ameen et al.^37^	Kingdom of Saudi Arabia	Severe sepsis and septic shock	Survivor	22 (12)	59 (5)	APACHE = 25 (4)	0
			Nonsurvivor	17 (9)	64 (4)	APACHE = 21 (2)	17 (100)
Beishuizen et al.^13^	The Netherlands	Healthy control		41 (23)	62 (9)	NA	0
		Multiple trauma		8 (7)	52 (17)	APACHE II = 10 (2)	0
		Septic shock		32 (20)	64 (13)	APACHE II = 15 (6)	11 (34)
			Survivor	21 (NR)	61 (11)	APACHE II = 12 (5)	0
			Nonsurvivor	11 (NR)	67 (14)	APACHE II = 18 (5)	11 (100)
			Without ARDS	24 (NR)	59 (13)	APACHE II = 11 (6)	NR
			With ARDS	8 (NR)	64 (12)	APACHE II = 19 (4)	NR
Bozza et al.^29^	Brazil	Healthy control		11 (NR)	NR	NA	NR
		Sepsis		17 (10)	59 (23)	APACHE II = 17 (6)	3 (18)
		Septic shock		25 (15)	59 (27)	APACHE II = 21 (7)	13 (52)
Brenner et al.^14^	Germany	Healthy control		18 (10)	35 (9)	NA	0
		Major surgery		28 (12)	62 (14)	NR	0
		Severe sepsis and septic shock	Survivor and nonsurvivor	87 (51)	69 (12)	NR	44 (51)
Calandra et al.^16^	Switzerland	Healthy control		6 (NR)	Median = 40	NA	NR
		Sepsis	Severe sepsis and septic shock	16 (13)	52 (18)	SAPS II = 45 (14)	6 (38)
Chuang et al.^38^	Taiwan	Severe sepsis	Survivor	81 (44)	67 (23)	APACHE II = 23 (8)	0
			Nonsurvivor	31 (24)			31 (100)
Chuang et al.^33^	Taiwan	Severe sepsis and septic shock	Survivor	109 (68)	71 (15)	APACHE II = 22 (8)	0
			Died in 48 h	12 (6)	68 (18)	APACHE II = 27 (7)	12 (100)
			Died after 48 h	32 (21)	74 (12)	APACHE II = 25 (8)	32 (100)
de Mendonca-Filho et al.^35^	Brazil	Sepsis	Negative microbiology	24 (16)	70 (2)	APACHE II = 15 (1)	5 (21)
			Positive microbiology	25 (17)	71 (2)	APACHE II = 16 (1)	12 (48)
Emonts et al.^31^	Switzerland and The Netherlands	Healthy control		196 (NR)	NR	NA	NR
		Sepsis, severe sepsis, and septic shock	Survivor	36 (18)	47 (17)	NR	0
			Early death	20 (17)	53 (14)	NR	20 (100)
			Late death	12 (9)	61 (13)	NR	12 (100)
Gando et al.^28^	Japan	Healthy control		10 (NR)	NR	NA	NR
		SIRS and sepsis	Without DIC	28 (17)	56 (3)	APACHE II = 17 (1)	1 (4)
			With DIC	20 (8)	51 (5)	APACHE II = 27 (2)	12 (60)
Gao et al.^17^	USA	Healthy control		53 (NR)	NR	NA	NR
		Sepsis		36 (NR)	NR	NR	NR
		Sepsis-induced acute lung injury		53 (NR)	NR	NR	19 (36)
Kofoed et al.^32^	Denmark	Healthy control		10 (NR)	NR	NA	NR
		Sepsis		10 (NR)	NR	NR	NR
Leaver et al.^25^	UK	Healthy control		20 (10)	NR	NA	NR
		Severe sepsis and septic shock		35 (22)	62 (22)	19 (6)	10 (29)
Lehmann et al.^12^	Germany	Healthy control		10 (NR)	NR	NA	NR
		Nonseptic critically ill		18 (17)	60 (18)	SOFA = 2 (1)	NR
		Severe sepsis		19 (14)	44 (16)	SOFA = 10 (2)	NR
Lehmann et al.^18^	Germany	Healthy control		34 (NR)	NR	NA	NR
		Nonseptic critically ill		10 (7)	61 (17)	SOFA = 3 (1)	0
		Severe sepsis	Survivor	23 (NR)	55 (11)	SOFA = 9 (3)	0
			Nonsurvivor	14 (NR)		SOFA = 16 (3)	14 (100)
Meawed et al.^15^	Egypt	Nonseptic systemic inflammation		28 (19)	50 (5)	NR	NR
		Sepsis	Survivor and nonsurvivor	25 (15)	53 (6)	APACHE II = 17 (3)	4 (16)
		Severe sepsis		27 (16)	63 (7)	APACHE II = 20 (3)	15 (56)
Merk et al.^26^	Canada	Healthy control		85 (NR)	NR	NA	NR
		Severe sepsis and septic shock		37 (22)	60 (17)	APACHE II = 22 (7)	10 (27)
Miyauchi et al.^34^	Japan	Sepsis	Normal adrenal response	22 (14)	63 (17)	APACHE II = 26 (6)	6 (27)
			Adrenal insufficiency	19 (16)	66 (15)	APACHE II = 26 (10)	6 (32)
Payen et al.^36^	France	Severe sepsis and septic shock	Without acute kidney injury	47 (30)	Median = 60	Median SOFA = 5	6 (12)
			Mild acute kidney injury	75 (47)	Median = 61	Median SOFA = 7	20 (26)
			Severe acute kidney injury	54 (34)	Median = 63	Median SOFA = 10	22 (41)
Pohl et al.^30^	Germany	Healthy control		10 (NR)	NR	NA	NR
		Nonseptic critically ill		42 (28)	69 (13)	APACHE II = 24 (9)	35 (83)
		Severe sepsis and septic shock		30 (19)	69 (11)	APACHE II = 26 (9)	13 (43)
Wiersinga et al.^27^	Thailand	Healthy controls		32 (23)	41 (9)	NA	NR
		Sepsis	Survivor and nonsurvivor	34* (17)	52 (16)	NR	15* (44)

*MIF levels were reported for 29 septic and 10 survivor patients.

*ARDS* adult respiratory distress syndrome, *APACHE* acute physiology and chronic health evaluation score, *DIC* disseminated intravascular coagulation, *NA* not applicable, *NR* not reported, *SAPS* simplified acute physiology score, *SIRS* systemic inflammatory response syndrome, *SOFA* sequential (sepsis-related) organ failure assessment score.

According to our quality assessment, 16 studies were considered as high quality, while 5 studies as moderate quality (Supplementary Table S2). Based on visual inspection of the funnel plots (Supplementary Figs. S1–S4), some asymmetry could be present, indicating the possible existence of publication bias, which was confirmed by the results of Egger’s test (*p* < 0.1) in one of the analyses (Supplementary Fig. S4). Sensitivity analysis was performed for overall SMD presented in the forest plots. The overall SMDs did not vary substantially after excluding any individual study, indicating that the results were not driven by one of the analyzed individual studies (Tables S3–S6).

### Blood levels of MIF in sepsis, noninfectious systemic inflammation, and healthy control groups

**Figure 2:**
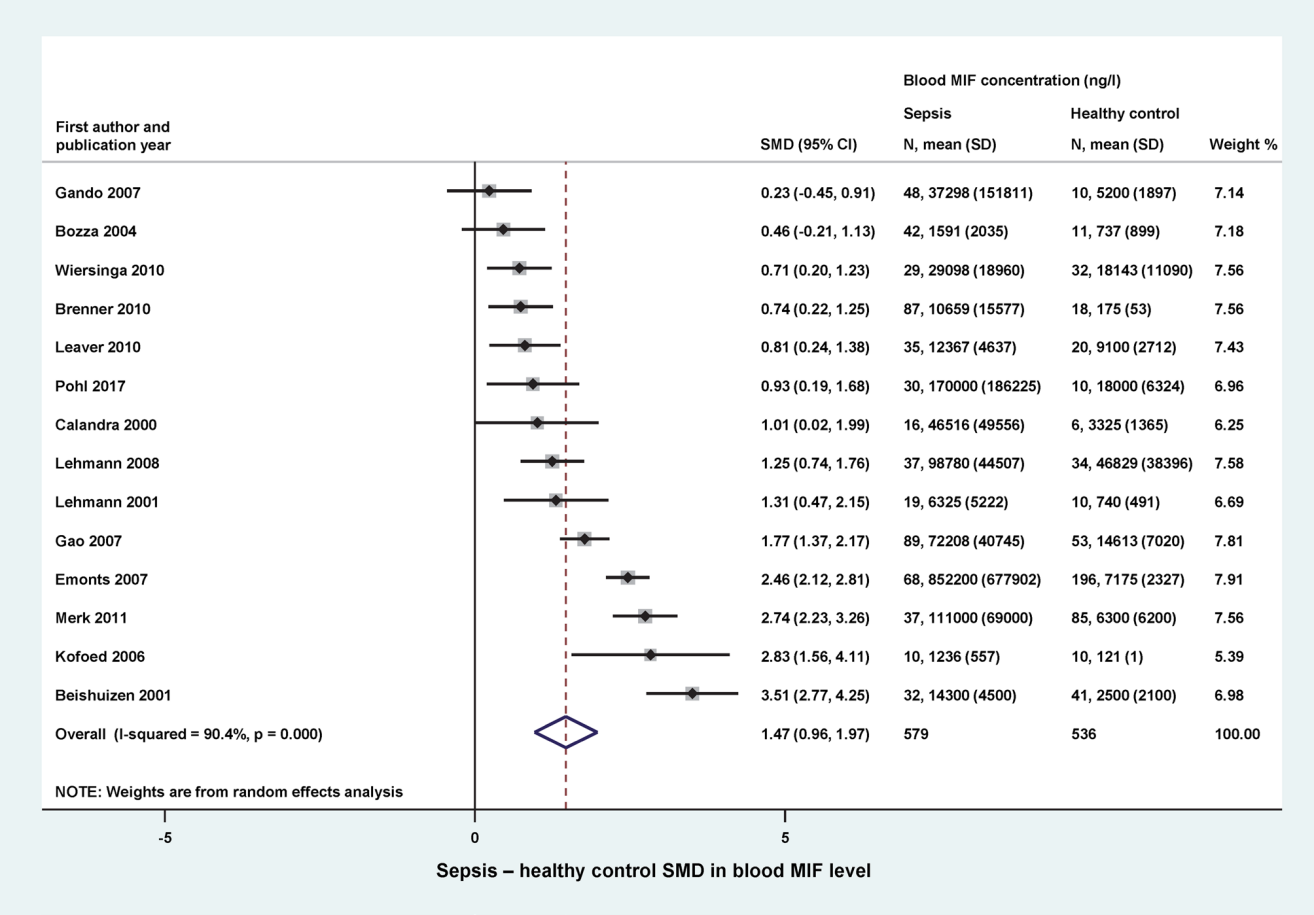
Forest plot of standardized mean differences (SMDs) in blood levels of macrophage migration inhibitory factor (MIF) between septic patients and healthy controls. Here, and in Figs. 3, 5, and 6 black diamonds represent the SMD for each study, while the left and right horizontal arms of the diamonds indicate the corresponding 95% confidence intervals (CIs). The size of the gray box surrounding the diamond is proportional to the relative weight of the study. The open rhombus on the bottom represents the average SMD calculated from the SMDs of all individual studies. The left and right vertices of the rhombus represent the CIs of the average SMD, while the vertical diagonal and the dashed line indicate the average SMD of all studies in the forest plot. A negative SMD indicates higher MIF levels in healthy controls, whereas an SMD greater than zero indicates increased MIF levels in sepsis. *SD* standard deviation.

First, we investigated the change in blood MIF levels in response to sepsis compared to healthy control subjects. We found 14 studies, reporting data from 579 septic patients and 536 healthy participants that could be included in our analysis (Fig. 2). The relative weight of the studies was similar, ranging between 5 and 8%. As it could be expected based on the function of MIF as a proinflammatory cytokine^11^, the blood levels of MIF were higher in septic patient groups than in controls in the analyzed studies with SMDs ranging from 0.23 to 3.51 between the groups. Overall, in septic patient groups blood MIF levels were significantly (*p* < 0.001) higher than in healthy controls with an SMD of 1.47 (95% CI: 0.96–1.97) (Fig. 2).

**Figure 3:**
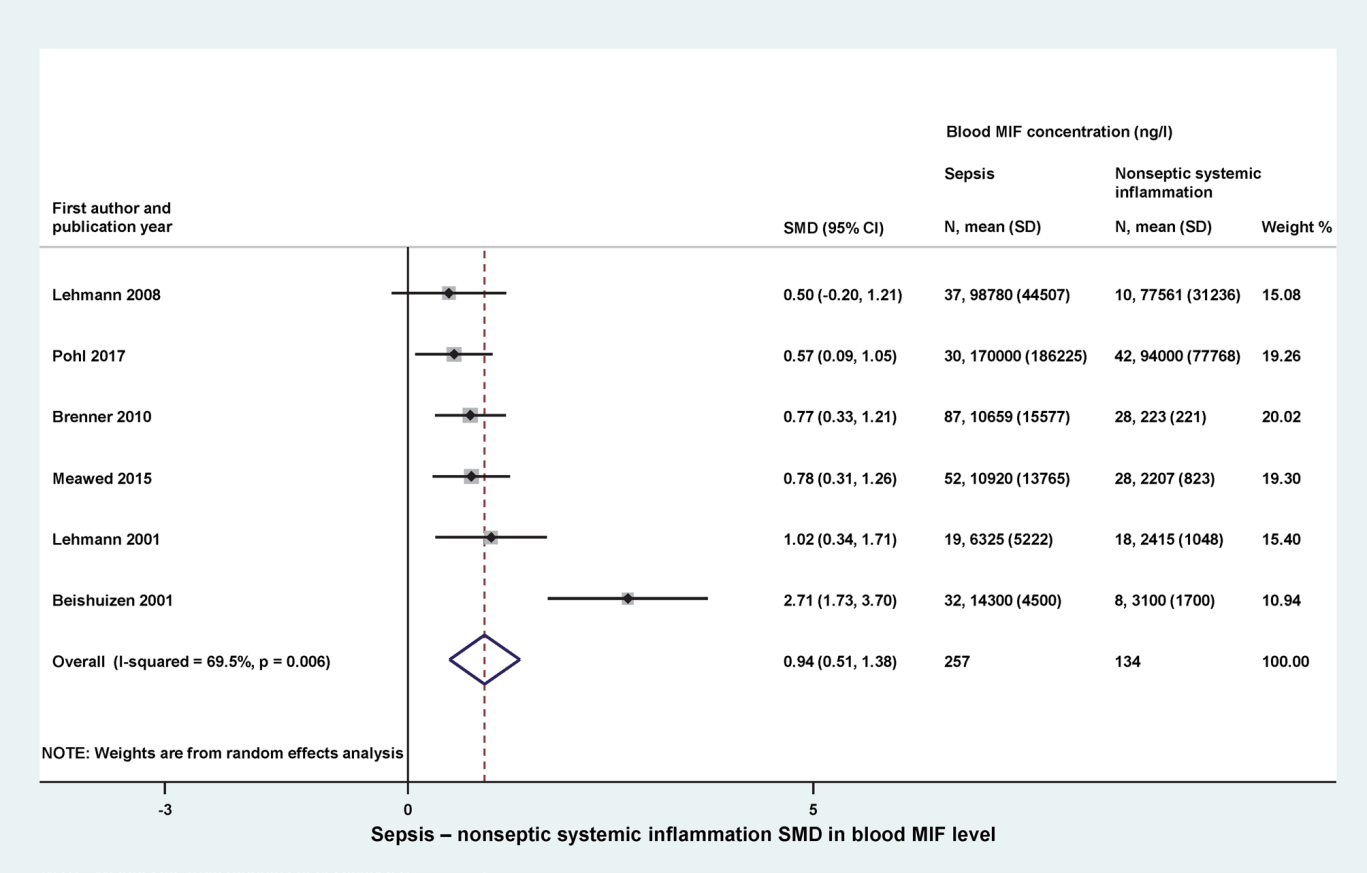
Forest plot of standardized mean differences (SMDs) in blood levels of macrophage migration inhibitory factor (MIF) between septic patients and patients with systemic inflammation due to noninfectious causes. *CI* confidence interval, *SD* standard deviation.

Next, we studied whether blood MIF levels are increased to a similar or to a different extent in sepsis and in noninfectious systemic inflammation. We could include 6 studies in the quantitative analyses, which reported data from 257 septic patients and 134 patients with nonseptic systemic inflammation (Fig. 3). In the latter group, the cause of systemic inflammation was surgical intervention^12,14,18^, multiple trauma^13^, and not sepsis-related fever^15^ or critical illness^30^ (see also Table 1). The relative weight of the studies ranged from 11 to 20%. Blood MIF levels were higher in septic patient groups than in patient groups with nonseptic systemic inflammation in all of the analyzed studies. The overall SMD was 0.94 (95% CI: 0.51–1.38) between the groups (*p* < 0.001) (Fig. 3).

**Figure 4:**
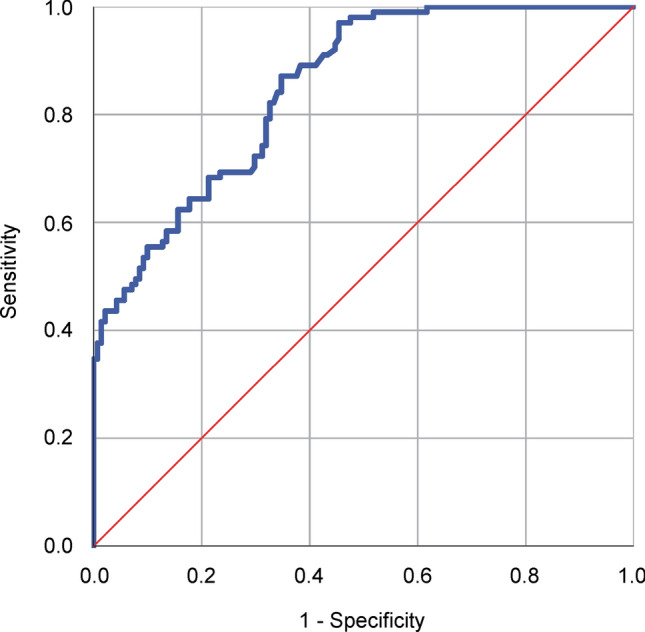
Receiver operating characteristic (ROC) curve analysis of the diagnostic performance of blood macrophage migration inhibitory factor (MIF) levels in sepsis. The individual data of septic patients (N = 101) and healthy controls (N = 141) were extracted from previously published studies^25–27^. The area under the blue ROC curve was 0.850. The diagonal red line serves as a reference line corresponding to the ROC curve of a diagnostic test that randomly classifies the condition (i.e., a test that has no diagnostic ability).

From three studies which presented blood MIF level values of individual participants^25–27^, we could extract the data of 101 septic patients and 141 healthy controls. ROC curve analysis of these data revealed an AUC of 0.850 (Fig. 4), indicating that blood MIF level measurement shows good sensitivity and specificity for the diagnosis of sepsis.

### Blood levels of MIF in septic patient groups with different severities of the disease

After studying MIF as a potential diagnostic biomarker in sepsis, we also wanted to analyze whether the elevation in blood MIF levels can predict the severity of the disease. We found eligible data to address this question from two approaches: (1) by comparing patient groups with less and more severe sepsis (e.g., based on the presence of organ dysfunction) within the same study; and (2) by comparing survivor and nonsurvivor septic patient groups within the same study.

**Figure 5:**
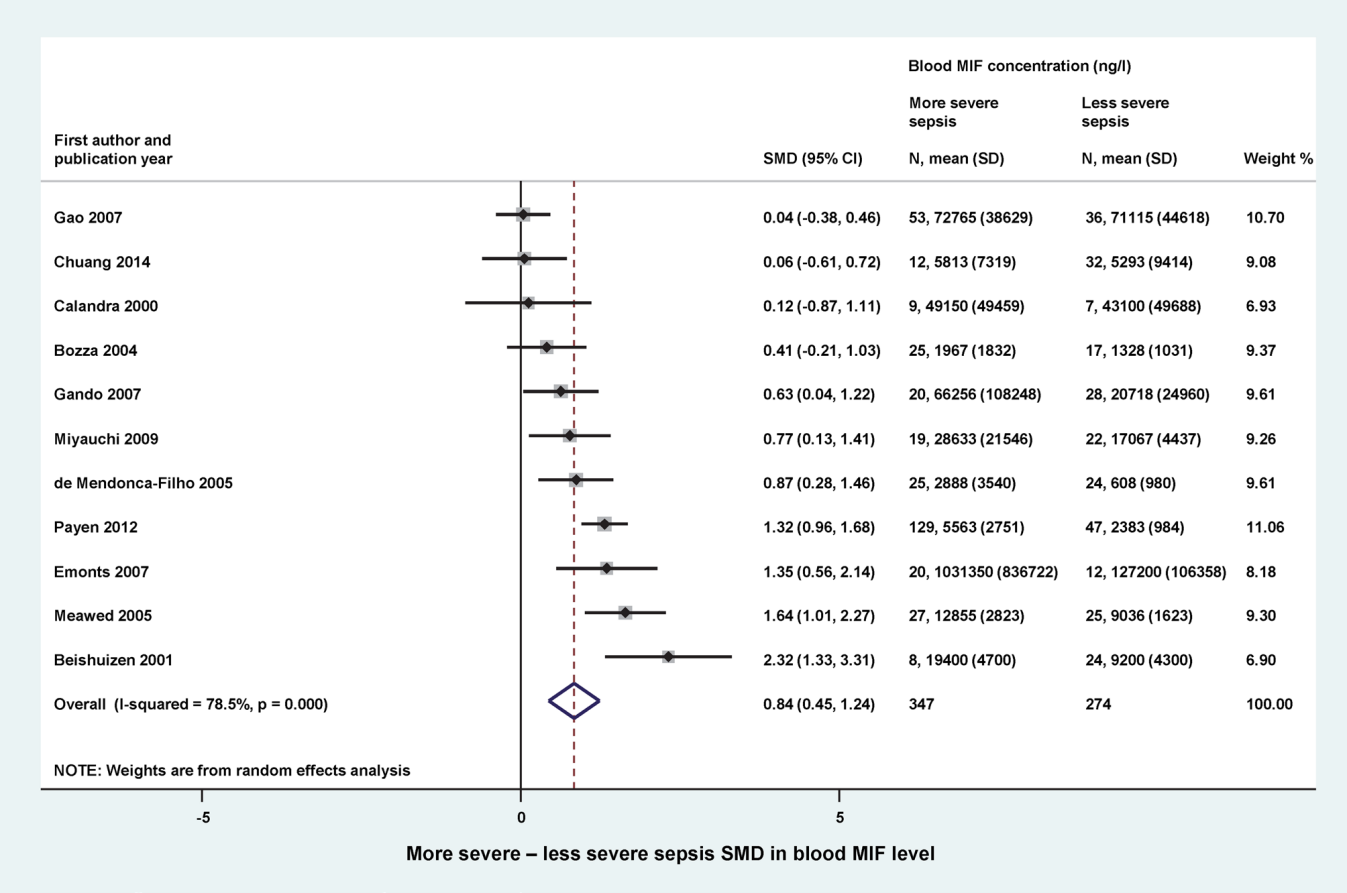
Forest plot of standardized mean differences (SMDs) in blood levels of macrophage migration inhibitory factor (MIF) between patients with more severe and less severe forms of sepsis. *CI* confidence interval, *SD* standard deviation.

We found 11 studies, in which blood MIF levels were reported in different severity groups of sepsis. The groups with more severe form of the disease were categorized based on different criteria in the different studies, which included the presence of one of the following conditions: organ damage (viz., pulmonary, kidney or adrenal gland dysfunction)^13,17,34,36^, septic shock^16,29^, early fatality^31,33^, severe sepsis^15^, disseminated intravascular coagulopathy^28^, and positive blood culture^35^ (for details, see Table 1). In the majority of the studies, higher clinical severity scores were also reported in the patient groups with more severe disease. In total, 347 patients were included in the more severe and 274 patients in the less severe septic groups. The relative weight of the studies was between 7 and 11%. Our meta-analysis revealed that blood MIF level was significantly (*p* < 0.001) higher in the more severe forms of sepsis with an overall SMD of 0.84 (95% CI: 0.45–1.24) (Fig. 5).

**Figure 6:**
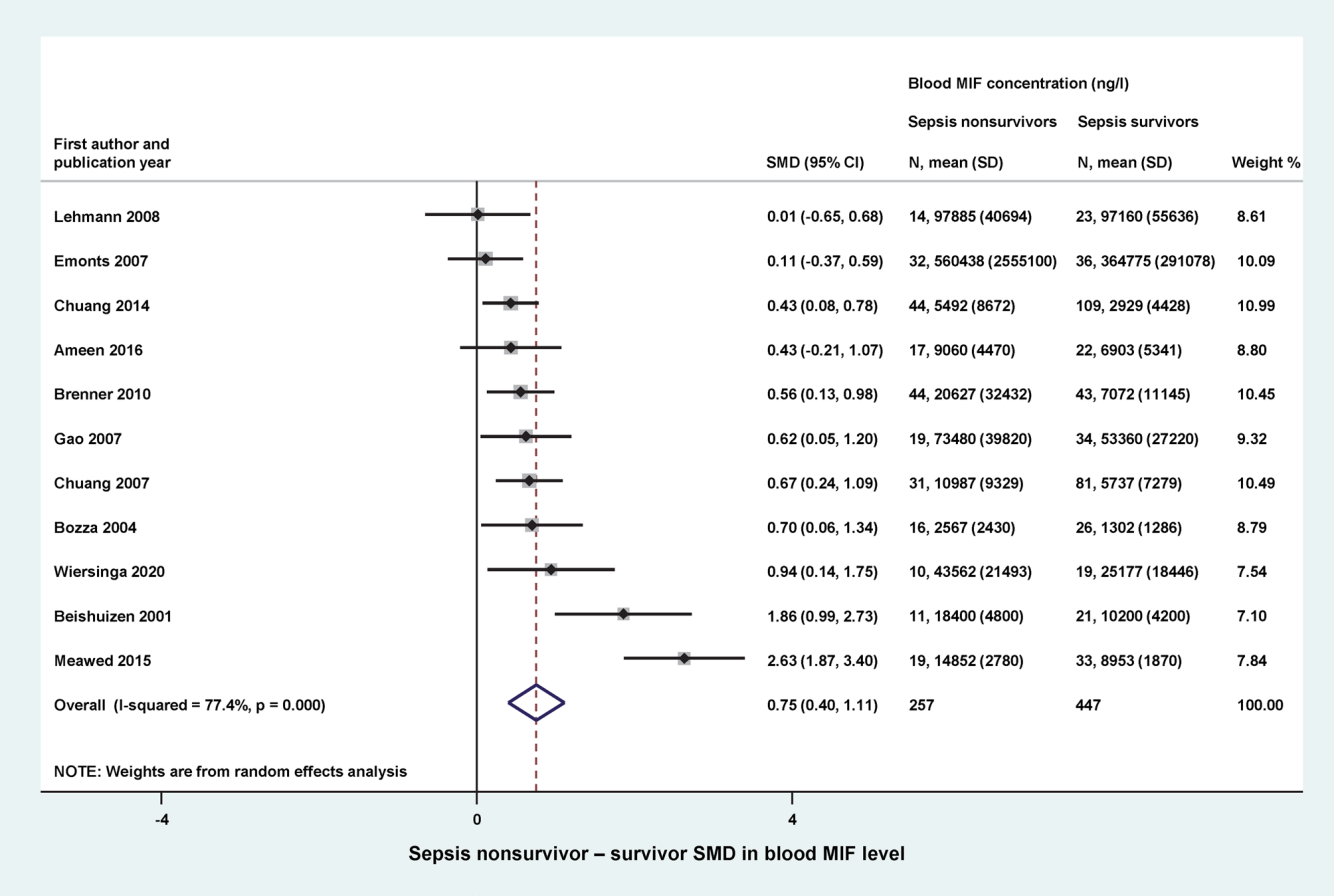
Forest plot of standardized mean differences (SMDs) in blood levels of macrophage migration inhibitory factor (MIF) between sepsis nonsurvivors and survivors. *CI* confidence interval, *SD* standard deviation.

Blood MIF levels were compared between survivors and nonsurvivors of sepsis in 11 studies, including 447 and 257 patients in the groups, respectively. The studies had similar relative weights, ranging from 7 to 11%. For the meta-analysis, SMD was calculated by subtracting the mean blood MIF level of sepsis survivors from that of sepsis nonsurvivors. We found that the overall SMD was significantly (*p* < 0.001) higher than zero (0.75, 95% CI: 0.40–1.11) (Fig. 6), indicating that blood MIF levels were markedly higher in nonsurvivors than in survivors of sepsis.

## Discussion

In the present study, we show that blood MIF level can be a useful biomarker in sepsis for both diagnostic and prognostic purposes, to the best of our knowledge for the first time, with the meta-analysis of the available data in the literature. The main new findings of our meta-analyses are that blood MIF levels are increased to a greater extent in sepsis than in systemic inflammation of noninfectious origins and that MIF levels are higher in the more severe forms of sepsis and in nonsurvivors than in less severe forms and survivors, respectively.

Sepsis affects tens of millions of patients annually and it constitutes an ongoing challenge for the healthcare system due to its high mortality and economic burden, especially in its severe forms^39^. A recent analysis showed that in intensive care units, hospital-acquired sepsis is frequent and accounts for a high (over 40%) mortality rate^40^. In order to improve outcomes, it is required to further develop the approaches for early diagnosis and implementation of adequate treatment of sepsis. The use of biomarkers can help to achieve these goals. As a consequence, a plethora of potential biomarkers was evaluated for the diagnosis and prognosis of sepsis (for a recent review, see^6^).

As an early step in the development of systemic inflammation, the activation of innate immune cells leads to the production of inflammatory cytokines^41^. MIF is one of these proinflammatory cytokines, which was originally thought to be produced in the pituitary gland and T lymphocytes, but later it was found to be expressed in a variety of cells, including endothelial cells, eosinophils, and macrophages^42^. Upon stimulation by endotoxins and cytokines, macrophages release MIF, which acts in concert with other cytokines (e.g., tumor necrosis factor-α) and promotes the acute inflammatory response^43^. In humans, high MIF concentrations were first found in the alveolar airspaces of patients with acute respiratory distress syndrome^44^, which is a frequent complication in severe (often fatal) forms of sepsis^45^. Since then, several studies showed that blood MIF level is increased in different forms of systemic inflammation^13,16,26^. As a consequence, MIF was considered amongst the potential diagnostic and prognostic biomarkers in sepsis^6,7,46^.

It has not been fully clarified, however, whether septic and nonseptic systemic inflammation can be distinguished based on the different extent of elevation in blood MIF levels. Some authors found that MIF levels were higher in sepsis than in noninfectious systemic inflammation^13–15,30^, whereas others did not find a significant difference in MIF levels between the two forms of systemic inflammation^12,18^. In the present study, we compared MIF levels in sepsis and in noninfectious inflammation of different origins (see Table 1, for details) in 257 and 134 patients, respectively, and showed that blood MIF concentration is markedly increased in case of sepsis compared to nonseptic systemic inflammation. These findings suggest that MIF can be used as a diagnostic tool to distinguish sepsis from other systemic inflammatory diseases. It can be assumed that the production of MIF is more enhanced when the triggering agent of the inflammatory reaction is a microbial pathogen than when it is a damage-associated molecular pattern (DAMP). Indeed, it has been shown that DAMPs and pathogen-associated molecular patterns (PAMPs) activate the immune system differently, in particular, DAMPs produce weaker innate immune activation than PAMPs, which also involves more pronounced production of inflammatory cytokines in case of PAMPs^47^. In line with these findings in experimental models, the increased MIF levels in multiple trauma patients were further elevated when an infection developed, suggesting that MIF may be an indicator of secondary infection^48,49^.

The prognostic value of MIF is also a controversial issue. In the study by Beishuizen et al.^13^, MIF levels tended to be higher in septic shock patients who developed acute respiratory distress syndrome than in those who did not (*p* = 0.115). MIF levels seemed higher in septic shock than in severe sepsis in the fundamental study by Calandra et al*.*^16^, but the difference between the groups was not significant. Furthermore, MIF levels did not differ between survivors and nonsurvivors of severe sepsis^18^, contradicting earlier reports about higher circulating MIF levels in nonsurvivor sepsis patients^13,14,50^, and about its association with fatal outcome in sepsis^29^. In the present work, we showed that MIF levels were significantly higher in the groups with worse prognosis, indicating that MIF can be a useful biomarker to predict the severity and the outcome of the disease. It can be assumed that in severe forms of sepsis an overt inflammatory reaction develops, which also involves a pronounced cytokine storm and excessive production of MIF. As a result, the pro- and anti-inflammatory processes become unbalanced, the inflammatory response loses its adaptive biological function, and turns into an unregulated, destructive process, which is no longer beneficial, but instead harmful for the host. The role of MIF can be crucial in the disruption of the pro- and anti-inflammatory balance, because MIF counter-regulates the anti-inflammatory and immunosuppressive effects of glucocorticoids^51–53^. Based on this scenario, it can be also understood, why neutralization of MIF with antibodies improved the outcome in animal models of severe systemic inflammation^16,54,55^. Whether MIF can be used as a therapeutic target and marker in septic patients, as proposed by different authors^16,56^, remains subject for future research.

Some limitations of our study should be noted. Due to the nature of the meta-analysis method, we have studied the reported mean MIF levels in patient groups, instead of MIF levels in individual patients. The latter approach would certainly allow one to draw firmer conclusions about the association between MIF and the diagnosis and prognosis of sepsis, but that would require access to the original data of the analyzed articles, which was not feasible. Due to lack of data, we could not perform a network meta-analysis to compare the performance of MIF with other frequently used inflammatory biomarkers, hence we cannot make any comment on its real value compared to others.

In our study, we compared blood MIF level in septic patients to that of either healthy controls or patients with nonseptic systemic inflammation. This method can be useful to identify potential diagnostic biomarkers, but it cannot be used to determine the diagnostic performance of MIF. To evaluate diagnostic test performance, the pre- and post-test probabilities are required, but “pre-test probability” amongst healthy controls is 0 (and thus “post-test probability” is also 0). The diagnostic performance of MIF is likely to be lower when distinguishing noninfectious systemic inflammation from sepsis, because of the smaller sample size (391 vs. 1115) and the lower SMD (0.94 vs. 1.47) compared to the analysis of healthy controls and septic patients.

An ideal study would include patients who were clinically suspected of sepsis, and compare their MIF levels with confirmed diagnosis of sepsis as this would allow assessment of the post-test probability of this test. Unfortunately, the analyzed studies did not have such ideal design. There were only two studies which included patients with suspicion of sepsis^29,30^, but those did not report the diagnostic performance of MIF only its good performance for the prediction of mortality. In another study, MIF levels between septic patients and healthy volunteers were compared and ROC curve analysis was performed, which indicated excellent sensitivity and specificity for MIF (AUC of 0.99)^26^. Further, in patients with clinical diagnosis of sepsis, MIF levels showed good performance in the prediction of positive bacterial cultures (AUC of 0.823)^35^.

For the assessment of diagnostic performance, the separation between positive and negative cases is important as it indicates the potential for false positive and false negative results. This is best assessed by ROC curve analysis, which requires individual patient data. As an attempt to perform ROC curve analysis, we extracted individual patient data from eligible papers^25–27^, and showed that blood MIF level has good diagnostic performance to distinguish septic patients from healthy controls. However, we could not collect sufficient data to perform the ROC curve analysis for the diagnostic value of MIF between infectious and noninfectious systemic inflammation and for its prognostic performance. Therefore, to exclude the possibility that mean levels of MIF simply differed significantly between the cohorts examined, in future studies additional ROC curve analyses are warranted to support our findings about the diagnostic and prognostic ability of MIF.

Another important issue with the comparison between sepsis and nonseptic systemic inflammation is that in 3 of the analyzed studies^12,13,18^ the clinical severity scores were significantly higher in septic than in nonseptic patients. Since we also showed that blood MIF levels are higher in more severe forms of sepsis than in less severe forms (Fig. 5), it cannot be excluded that the difference in MIF levels between septic and nonseptic patients was also influenced by the higher severity scores in the septic patients in some of the studies.

The studied population of patients was quite diverse and statistical, methodological, and medical differences in study design could all contribute to the considerably high between-study heterogeneity (indicated by an *I*^2^ of 70–90%), as observed in our analysis (Figs. 2, 3, 5, 6). To account for the presence of heterogeneity, we used the random-effects model in all forest plots of our meta-analyses.

In the analyzed studies, blood MIF levels between patients’ groups were compared within the same study and the difference was included in the forest plot. Since the reported MIF values differed substantially among the analyzed studies, ranging between 121 ng/l^32^ and 46,829 ng/l^18^ in healthy controls (Fig. 2), SMDs had to be used to mitigate methodological differences in MIF level measurements. Consequently, in the present analysis we could not determine a specific cut-off MIF level which would be a diagnostic or prognostic threshold in sepsis. The most convincing method to obtain direct evidence for the diagnostic and prognostic performance of MIF in sepsis would be to conduct high-quality, targeted clinical trials in a broad population of patients who are clinically suspected of sepsis. Until such or similar trials are conducted, we are restricted to use different (not so direct) approaches, e.g., meta-analyses. In the design of future studies, other classical and novel biomarkers, perhaps in combination with MIF, may be also considered, for example, neutrophil CD64, which was superior to procalcitonin for the identification of sepsis according to a recent meta-analysis^57^.

Despite the mentioned limitations, we believe that the size of the analyzed sample (N = 1876) was big enough to mitigate the methodological differences among the studies, therefore we may draw, at least some, conclusions about the potential diagnostic and prognostic value of MIF in septic patients.

## Conclusions

To the best of our knowledge, this is the first meta-analysis to show that blood MIF levels could have diagnostic ability to differentiate between infectious and noninfectious systemic inflammation and could have prognostic value for the outcome of sepsis. Our results can also serve as an encouraging basis for the design of high-quality, targeted clinical studies aiming to determine the real diagnostic and prognostic performance of MIF level measurements in sepsis.

## Supplementary Information

Supplementary Information 1.

## Data Availability

All data generated or analyzed during this study are included in this published article and its supplementary information.
