# Supplementary material for: Macrophage migration inhibitory factor as a diagnostic and predictive biomarker in sepsis: meta-analysis of clinical trials
Source: Sci Rep. 2021 Apr 13;11:8051. doi: 10.1038/s41598-021-87613-0 (PMC8044150; doi:10.1038/s41598-021-87613-0)
Supplement: Supplementary file 1 — Supplementary Information 1. [file 41598_2021_87613_MOESM1_ESM.pdf]

SUPPLEMENTARY INFORMATION

Macrophage migration inhibitory factor as a diagnostic and predictive biomarker in sepsis: meta-analysis of clinical trials

Janos Toldi, David Nemeth, Peter Hegyi, Zsolt Molnar, Margit Solymar, Nelli Farkas, Hussain Alizadeh, Zoltan Rumbus, Eszter Pakai, and Andras Garami

Table of contents:

Table S1: PRISMA checklist.

Table S2: Quality assessment of the studies included in the meta-analysis using the Newcastle-Ottawa Scale.

Table S3: Sensitivity analysis of SMD of blood MIF levels between septic patients and healthy controls.

Table S4: Sensitivity analysis of SMD of blood MIF levels between septic patients and patients with systemic inflammation due to noninfectious causes.

Table S5: Sensitivity analysis of SMD of blood MIF levels between patients with more severe and less severe forms of sepsis.

Table S6: Sensitivity analysis of SMD of blood MIF levels between sepsis nonsurvivors and survivors.

Figure S1: Funnel plot of the studies that were included in the comparison of blood MIF levels between septic patients and healthy controls.

Figure S2: Funnel plot of the studies that were included in the comparison of blood MIF levels between septic patients and patients with systemic inflammation due to noninfectious causes.

Figure S3: Funnel plot of the studies that were included in the comparison of blood MIF levels between patients with more severe and less severe forms of sepsis.

Figure S4: Funnel plot of the studies that were included in the comparison of blood MIF levels between sepsis nonsurvivors and survivors.

TABLE S1

PRISMA checklist

Section/topic	#	Checklist item	Reported on page #
**TITLE**			
Title	1	Identify the report as a systematic review, meta-analysis, or both.	1
**ABSTRACT**			
Structured summary	2	Provide a structured summary including, as applicable: background; objectives; data sources; study eligibility criteria, participants, and interventions; study appraisal and synthesis methods; results; limitations; conclusions and implications of key findings; systematic review registration number.	1
**INTRODUCTION**			
Rationale	3	Describe the rationale for the review in the context of what is already known.	1-2
Objectives	4	Provide an explicit statement of questions being addressed with reference to participants, interventions, comparisons, outcomes, and study design (PICOS).	2
**METHODS**			
Protocol and registration	5	Indicate if a review protocol exists, if and where it can be accessed (*e.g.*, Web address), and, if available, provide registration information, including registration number.	2
Eligibility criteria	6	Specify study characteristics (*e.g.*, PICOS, length of follow-up) and report characteristics (*e.g.*, years considered, language, publication status) used as criteria for eligibility, giving rationale.	2
Information sources	7	Describe all information sources (*e.g.*, databases with dates of coverage, contact with study authors to identify additional studies) in the search and date last searched.	2
Search	8	Present full electronic search strategy for at least one database, including any limits used, such that it could be repeated.	2
Study selection	9	State the process for selecting studies (*i.e.*, screening) and list the inclusion and exclusion criteria.	2
Data collection process	10	Describe method of data extraction from reports (*e.g.*, piloted forms, independently, in duplicate) and any processes for obtaining and confirming data from investigators.	2
Data items	11	List and define all variables for which data were sought (*e.g.*, PICOS, funding sources) and any assumptions and simplifications made.	2
Risk of bias in individual studies	12	Describe methods used for assessing risk of bias of individual studies (including specification of whether this was done at the study or outcome level), and how this information is to be used in any data synthesis.	2
Summary measures	13	State the principal summary measures (*e.g.*, risk ratio, difference in means).	2
Synthesis of results	14	Describe the methods of handling data and combining results of studies, if done, including measures of consistency for each meta-analysis.	2
Risk of bias across studies	15	Specify any assessment of risk of bias that may affect the cumulative evidence (*e.g.*, publication bias, selective reporting within studies).	2
Additional analyses	16	Describe methods of additional analyses (*e.g.*, sensitivity or subgroup analyses, meta-regression), if done, indicating which were prespecified.	2-3

RESULTS			
Study selection	17	Give numbers of studies screened, assessed for eligibility, and included in the review, with reasons for exclusion at each stage, ideally with a flow diagram.	3; Fig. 1
Study characteristics	18	For each study, present characteristics for which data were extracted (*e.g.*, study size, PICOS, follow-up period) and provide the citations.	Table 1
Risk of bias within studies	19	Present data on risk of bias of each study and, if available, any outcome level assessment (see item 12).	Table S2
Results of individual studies	20	For all outcomes considered (benefits or harms), present, for each study: (a) simple summary data for each intervention group and (b) effect estimates and confidence intervals, ideally with a “forest plot”.	5; Figs. 2, 3, 5, and 6
Synthesis of results	21	Present results of each meta-analysis done, including confidence intervals and measures of consistency.	5; Figs. 2, 3, 5, and 6
Risk of bias across studies	22	Present results of any assessment of risk of bias across studies (see Item 15).	Figs. S1-S4
Additional analysis	23	Give results of additional analyses, if done [(*e.g.*, sensitivity or subgroup analyses, meta-regression (see Item 16)].	5; Fig. 4; Tables S3-S6
**DISCUSSION**			
Summary of evidence	24	Summarize the main findings, including the strength of evidence for each main outcome; consider their relevance to key groups (*e.g.*, healthcare providers, users, and policy makers).	6-10
Limitations	25	Discuss limitations at study and outcome level (*e.g.*, risk of bias), and at review-level (*e.g.*, incomplete retrieval of identified research, reporting bias).	9-10
Conclusions	26	Provide a general interpretation of the results in the context of other evidence, and implications for future research.	10
**FUNDING**			
Funding	27	Describe sources of funding for the systematic review and other support (*e.g.*, supply of data); role of funders for the systematic review.	12

TABLE S2

Quality assessment of the studies included in the meta-analysis using the Newcastle-Ottawa Scale

Study report	Selection	Comparability	Outcome / Exposure	Total score
Ameen et al. 2016	****	*	**	7
Beishuizen et al. 2001	***	**	**	7
Bozza et al. 2004	****	**	***	9
Brenner et al. 2010	***	**	**	7
Calandra et al. 2000	***	*	**	6
Chuang et al. 2007	****	*	**	7
Chuang et al. 2014	****	*	**	7
de Mendonca-Filho et al. 2005	****	**	**	8
Emonts et al. 2007	****	*	***	8
Gando et al. 2007	***	*	**	6
Gao et al. 2007	***	*	***	7
Kofoed et al. 2006	***	**	**	7
Leaver et al. 2010	****	**	**	8
Lehmann et al. 2001	***	**	**	7
Lehmann et al. 2008	***	**	**	7
Meawed et al. 2015	***	*	**	6
Merk et al. 2011	***	*	**	6
Miayuchi et al. 2009	****	*	**	7
Payen et al. 2012	***	**	**	7
Pohl et al. 2017	***	*	**	6
Wiersinga et al. 2010	***	**	**	7

A score of 7 to 9 indicates a good, a score of 4 to 6 a fair, and a score of 0 to 3 a low methodological quality.

TABLE S3

Sensitivity analysis of standardized mean difference (SMD) of blood macrophage migration inhibitory factor (MIF) levels between septic patients and healthy controls

Study omitted	Estimated SMD	95% Confidence interval	
Beishuizen et al. 2001	**1.3131386**	**0.83256066**	**1.7937164**
Bozza et al. 2004	**1.542878**	**1.0238485**	**2.0619075**
Brenner et al. 2010	**1.5268586**	**0.9980076**	**2.0557096**
Calandra et al. 2000	**1.4980285**	**0.97095335**	**2.0251036**
Emonts et al. 2007	**1.3799106**	**0.8725971**	**1.8872242**
Gando et al. 2007	**1.5613711**	**1.0528249**	**2.0699174**
Gao et al. 2007	**1.4433948**	**0.87894446**	**2.0078452**
Kofoed et al. 2006	**1.3892726**	**0.87214762**	**1.9063975**
Leaver et al. 2010	**1.5203255**	**0.98886555**	**2.0517855**
Lehmann et al. 2001	**1.4789149**	**0.94641781**	**2.0114119**
Lehmann et al. 2008	**1.4859716**	**0.93801826**	**2.0339251**
Merk et al. 2011	**1.3613919**	**0.85557604**	**1.8672078**
Pohl et al. 2017	**1.5072438**	**0.97631407**	**2.0381734**
Wiersinga et al. 2010	**1.5301822**	**1.0035641**	**2.0568004**
None	**1.4670072**	**0.9625479**	**1.9714665**

TABLE S4

Sensitivity analysis of standardized mean difference (SMD) of blood macrophage migration inhibitory factor (MIF) levels between septic patients and patients with systemic inflammation due to noninfectious causes

Study omitted	Estimated SMD	95% Confidence interval	
Beishuizen et al. 2001	**0.72372264**	**0.4889625**	**0.95848274**
Brenner et al. 2010	**1.0167089**	**0.4488349**	**1.5845827**
Lehmann et al. 2001	**0.94416475**	**0.43312234**	**1.4552072**
Lehmann et al. 2008	**1.0349311**	**0.5354175**	**1.5344447**
Meawed et al. 2015	**1.0095743**	**0.45152059**	**1.567628**
Pohl et al. 2017	**1.0511286**	**0.51861775**	**1.5836395**
None	**0.94433918**	**0.5105363**	**1.3781421**

TABLE S5

Sensitivity analysis of standardized mean difference (SMD) of blood macrophage migration inhibitory factor (MIF) levels between patients with more severe and less severe forms of sepsis

Study omitted	Estimated SMD	95% Confidence interval	
Beishuizen et al. 2001	**0.73298991**	**0.35420334**	**1.1117765**
Bozza et al. 2004	**0.88733292**	**0.45857477**	**1.3160911**
Calandra et al. 2000	**0.89526081**	**0.48256442**	**1.3079573**
Chuang et al. 2014	**0.91881663**	**0.50963479**	**1.3279985**
de Mendonca-Filho et al. 2005	**0.84071708**	**0.40133503**	**1.280099**
Emonts et al. 2007	**0.79637277**	**0.3768**	**1.2159455**
Gando et al. 2007	**0.8657974**	**0.42823938**	**1.3033555**
Gao et al. 2007	**0.93599939**	**0.56038755**	**1.3116113**
Meawed et al. 2015	**0.75772506**	**0.35516757**	**1.1602826**
Miayuchi et al. 2009	**0.85059726**	**0.41473499**	**1.2864596**
Payen et al. 2012	**0.78183091**	**0.36327559**	**1.2003863**
None	**0.84135154**	**0.44536898**	**1.2373341**

TABLE S6

Sensitivity analysis of standardized mean difference (SMD) of blood macrophage migration inhibitory factor (MIF) levels between sepsis nonsurvivors and survivors

Study omitted	Estimated SMD	95% Confidence interval	
Ameen et al. 2016	**0.79046631**	**0.4045704**	**1.1763623**
Beishuizen et al. 2001	**0.66525865**	**0.32198629**	**1.0085311**
Bozza et al. 2004	**0.76476264**	**0.37764108**	**1.1518843**
Brenner et al. 2010	**0.78628105**	**0.38203526**	**1.1905268**
Chuang et al. 2007	**0.77411079**	**0.36889201**	**1.1793295**
Chuang et al. 2014	**0.80348003**	**0.39395151**	**1.2130086**
Emonts et al. 2007	**0.82790214**	**0.45119721**	**1.2046071**
Gao et al. 2007	**0.77398223**	**0.3826614**	**1.1653031**
Lehmann et al. 2008	**0.82480216**	**0.45337233**	**1.196232**
Meawed et al. 2015	**0.55956095**	**0.3251034**	**0.79401851**
Wiersinga et al. 2010	**0.74225289**	**0.36428884**	**1.120217**
None	**0.75497058**	**0.40006185**	**1.1098793**

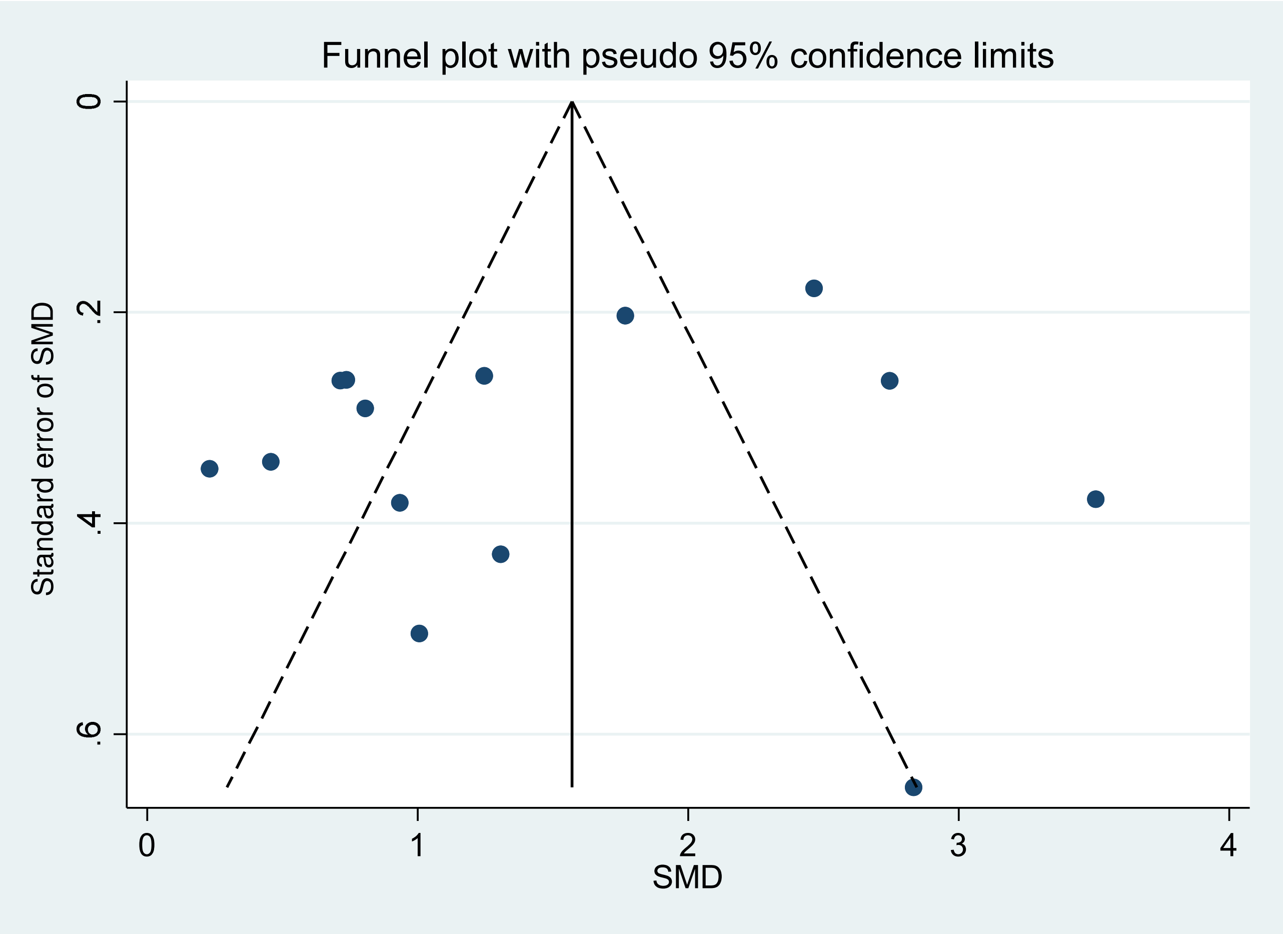

FIGURE S1. Funnel plot of the studies that were included in the comparison of blood macrophage migration inhibitory factor (MIF) levels between septic patients and healthy controls (n = 14, Egger's test: $p = 0.407$). Here, and in Figures S2-S4, the dots represent results from studies included in the corresponding forest plot. The triangular area is expected to harbor 95% of data in the absence of publication bias. The average standardized mean difference (SMD) for all studies corresponds to the "funnel axis" (vertical black line). A high degree of asymmetry in the distribution of data relative to the funnel axis and significant Egger's test ($p < 0.1$) indicate that the results are likely to be affected by a bias.

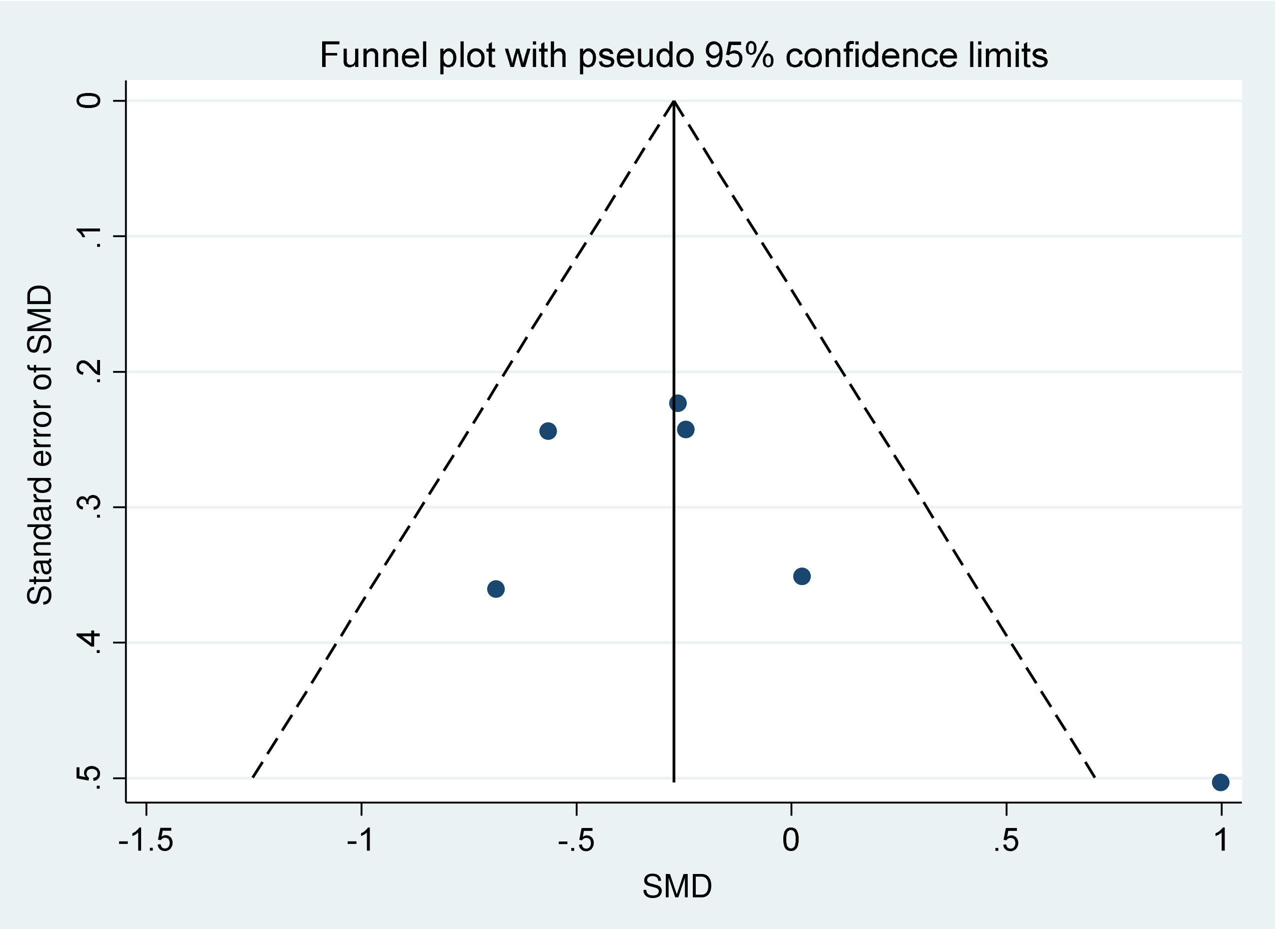

FIGURE S2. Funnel plot of the studies that were included in the comparison of blood macrophage migration inhibitory factor (MIF) levels between septic patients and patients with systemic inflammation due to noninfectious causes (n = 6).

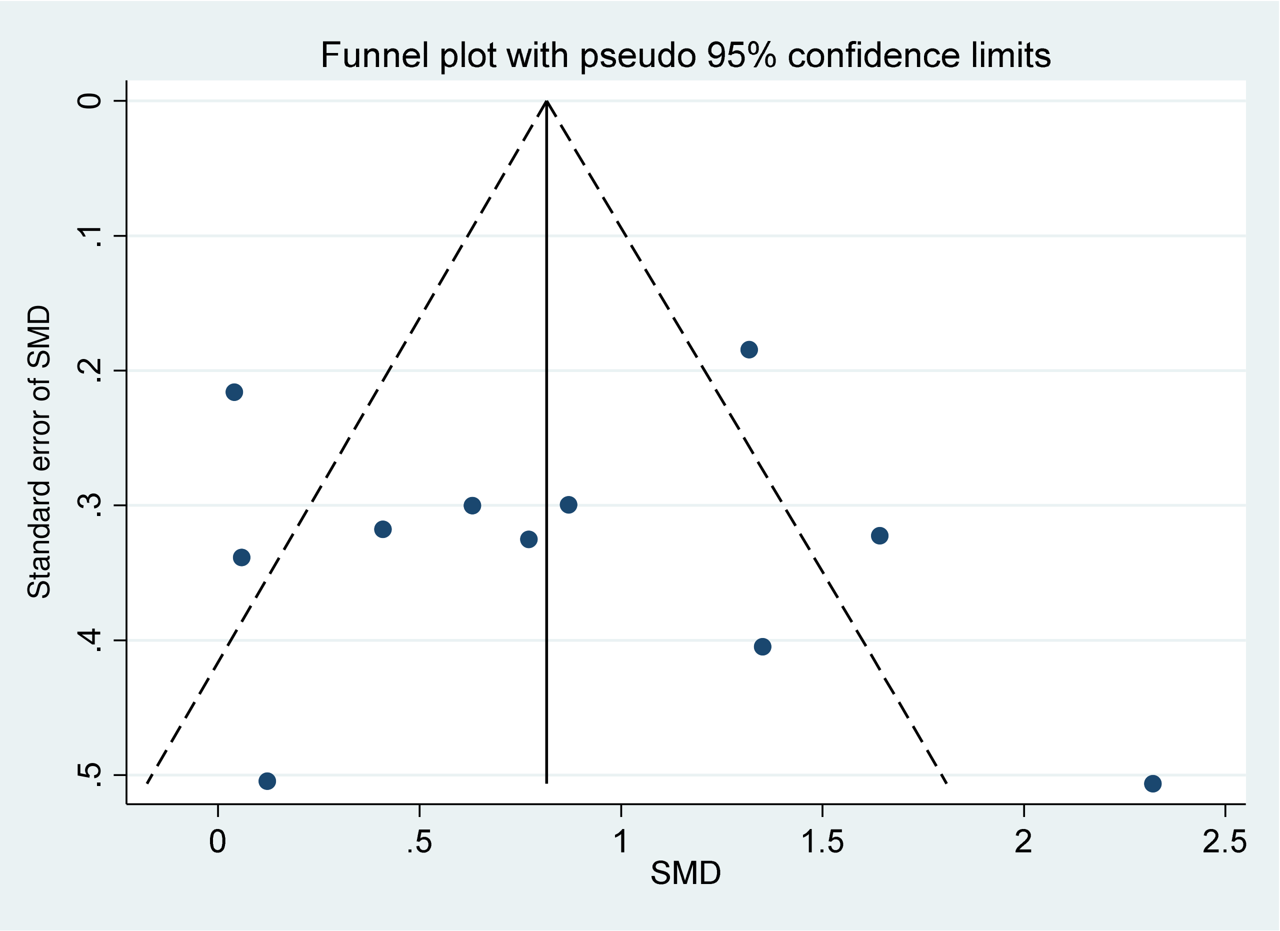

FIGURE S3. Funnel plot of the studies that were included in the comparison of blood macrophage migration inhibitory factor (MIF) levels between patients with more severe and less severe forms of sepsis (n = 11, Egger's test: $p = 0.815$).

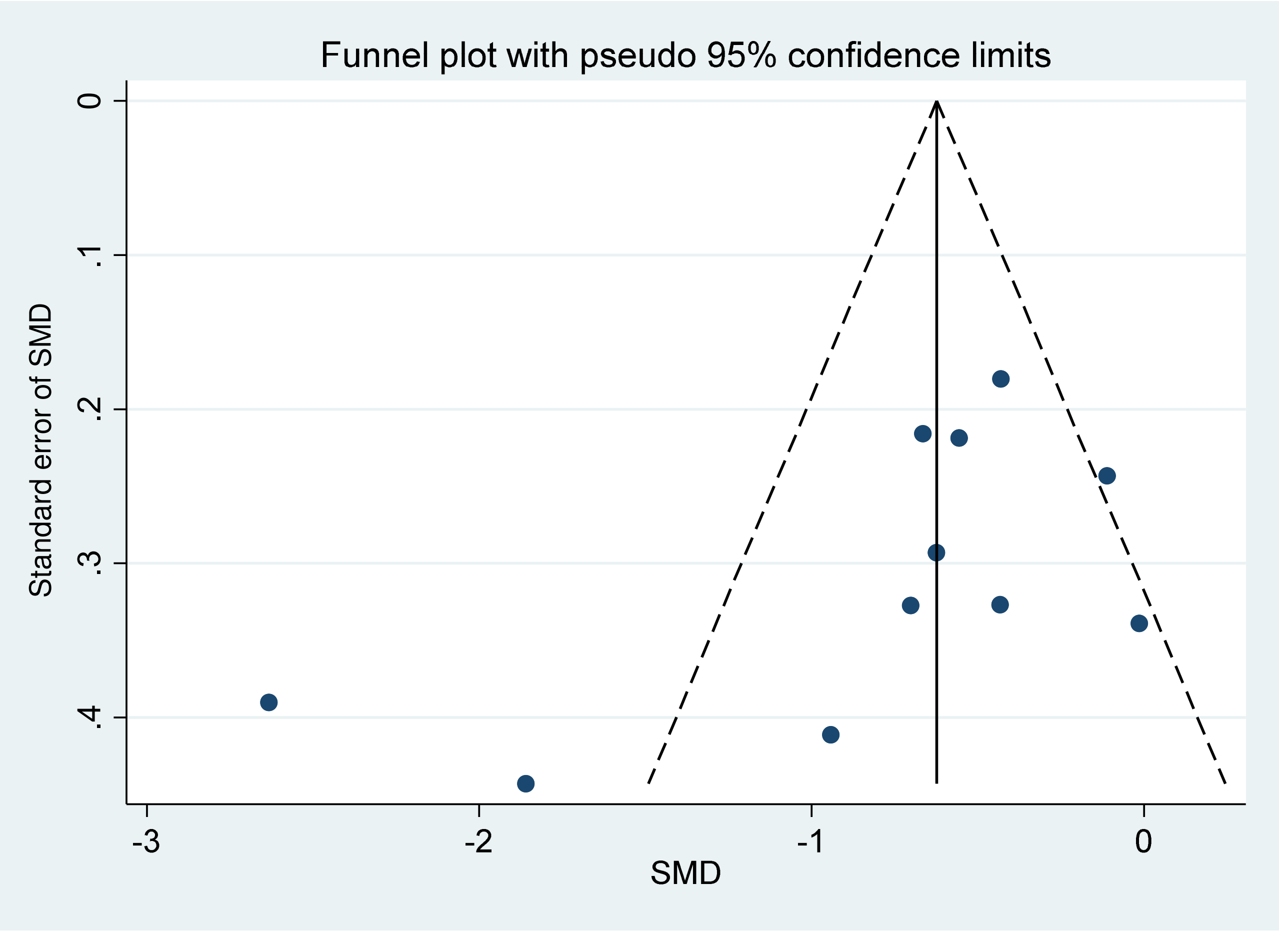

FIGURE S4. Funnel plot of the studies that were included in the comparison of blood macrophage migration inhibitory factor (MIF) levels between sepsis nonsurvivors and survivors (n = 11, Egger's test: $p = 0.086$).
